# CD28 deficiency leads to accumulation of germinal-center independent IgM^+^ experienced B cells and to production of protective IgM during experimental malaria

**DOI:** 10.1371/journal.pone.0202522

**Published:** 2018-08-27

**Authors:** Henrique Borges da Silva, Érika Machado de Salles, Eliana Faquim Lima-Mauro, Luiz Roberto Sardinha, José Maria Álvarez, Maria Regina D’Império Lima

**Affiliations:** 1 Departamento de Imunologia, Instituto de Ciências Biomédicas (ICB), Universidade de São Paulo (USP), São Paulo, Brazil; 2 Laboratório de Imunopatologia, Instituto Butantan, São Paulo, Brazil; 3 Instituto Israelita de Ensino e Pesquisa Albert Einstein, São Paulo, Brazil; Instituto Rene Rachou, BRAZIL

## Abstract

Protective immunity to blood-stage malaria is attributed to *Plasmodium*-specific IgG and effector-memory T helper 1 (Th1) cells. However, mice lacking the costimulatory receptor CD28 (CD28KO) maintain chronic parasitemia at low levels and do not succumb to infection, suggesting that other immune responses contribute to parasite control. We report here that CD28KO mice develop long-lasting non-sterile immunity and survive lethal parasite challenge. This protection correlated with a progressive increase of anti-parasite IgM serum levels during chronic infection. Serum IgM from chronically infected CD28KO mice recognize erythrocytes infected with mature parasites, and effectively control *Plasmodium* infection by promoting parasite lysis and uptake. These antibodies also recognize autoantigens and antigens from other pathogens. Chronically infected CD28KO mice have high numbers of IgM^+^ plasmocytes and experienced B cells, exhibiting a germinal-center independent Fas^+^GL7^-^CD38^+^CD73^-^ phenotype. These cells are also present in chronically infected C57BL/6 mice although in lower numbers. Finally, IgM^+^ experienced B cells from cured C57BL/6 and CD28KO mice proliferate and produce anti-parasite IgM in response to infected erythrocytes. This study demonstrates that CD28 deficiency results in the generation of germinal-center independent IgM^+^ experienced B cells and the production of protective IgM during experimental malaria, providing evidence for an additional mechanism by which the immune system controls *Plasmodium* infection.

## Introduction

Protection against clinical blood-stage malaria in humans and mice typically involves parasite-specific IgG antibody production [1][2]. Data from mouse malaria models suggest that production of these antibodies depends on CD4^+^ T cells and mostly occurs after control of acute infection [3][4]. Among the malaria mouse models, *Plasmodium chabaudi* (*Pc*) infection has been used to investigate the development of adaptive immunity due to its similarities to the human disease that is caused by *Plasmodium falciparum *[5]. The early CD4^+^ T cell response to *Pc* infection provides large amounts of pro-inflammatory cytokines and helps B cells to secrete polyclonal IgG [6][7]. However, *Pc*-infected mice also produce IgM in a T-cell independent manner [4][7]; IgM production is also observed in humans exposed to malaria [8][9], however a protective role for these antibodies is unclear [10]. Additionally, somatically hypermutated IgM+ memory B cells are found in both humans and mice infected with *Plasmodium *[11].

CD28 is a costimulatory molecule fundamental for the full development of CD4^+^ T cell responses [12] and CD4^+^ T cell-driven antibody class switch [13]. We previously showed that mice lacking CD28 do not eliminate chronic *Pc* parasitemia, due to the lack of memory CD4^+^ T cells and anti-parasite IgG [14]. However, despite the absence of full protective immunity, parasitemia in these mice persists at low levels during chronic infection, suggesting the contribution of other protective mechanisms. IgM participates in several immune effector mechanisms, such as complement system activation [15], antigen agglutination [16], dead and damaged cell scavenging [17] and lymphocyte activation through Fcμ receptors [18]. During encapsulated bacterial infections, IgM opsonizes bacilli, facilitates their removal by phagocytic cells and effectively combats the infection [19][20].

A full characterization of IgM produced in response to *Plasmodium* infection, as well as its potential anti-pathogenic roles have not been studied yet. We hypothesized that CD28KO mice would offer a good model to investigate the protective role of IgM against malaria given their deficiency in developing acquired immunity. The present study shows that CD28KO mice accumulated serum anti-parasite IgM in response to chronic parasitemia. The IgM response was associated with high numbers of IgM-producing plasmocytes and IgM^+^ experienced B cells in the spleen. Our results show that IgM produced in response to chronic parasitemia promotes parasite control in CD28KO mice, suggesting an additional antimalarial mechanism for protection against malaria.

## Results

### CD28KO mice develop long-lasting non-sterile protective immunity against blood-stage *Pc* malaria

**Fig 1:**
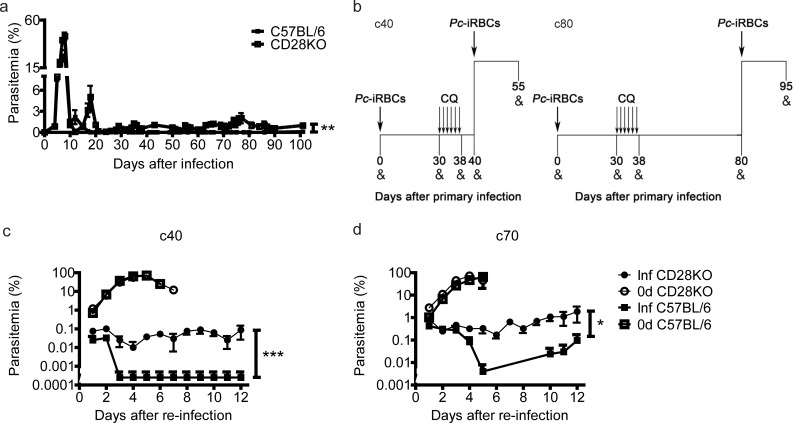
Parasitemia in C57BL/6 and CD28KO mice during primary and secondary *Pc* infections. (**a**) Parasitemia curves in mice infected intraperitoneally (i.p.) with 1 x 10^6^ *Pc*-iRBCs. (**b**) The experimental design for the secondary infections. Reminiscent parasitemia was eliminated by treating 31 to 38 days post-infection (p.i.) mice with chloroquine (CQ). At days 40 (c40) or 80 (c80) p.i., mice were challenged with 1 x 10^8^ *Pc*-iRBC. Experimental analyses were performed when indicated (&). (**c**) Parasitemia curves after secondary infection in c40 mice and age-matched controls (0d). (**d**) Parasitemia curves after secondary infection in c80 mice and age-matched controls (0d). In **a**, **c** and **d**, the significant differences (*p<0.05, **p<0.01, ***p<0.001) between the indicated groups are shown. Data from three independent experiments (n = 5–7, mean ± standard error mean—SEM) is shown.

In accordance with our previous study [14], CD28KO (*Cd28*^-/-^) mice infected with *Pc*-infected red blood cells (*Pc*-iRBCs) controlled the first parasitemia peak, but developed increased chronic parasitemia as defined by the presence of detectable parasitemia percentages in the circulating blood (i.e., above 0.1%) (Fig 1A). In C57BL/6 (*Cd28*^+/+^) mice, *Pc*-iRBCs were no longer detected by microscopic examination after clearance of acute parasitemia. Because generation of classic memory T and B cell responses to *Pc* infection requires CD28 signaling [14], it is intriguing how CD28KO mice survive acute infection and maintain relatively low levels of chronic parasitemia. To investigate whether this protection depends on parasite persistence, C57BL/6 and CD28KO mice at 30 days post-infection (p.i.) were submitted to a curative chloroquine treatment and then challenged with a lethal parasite dose at 40 or 80 days p.i. (c40 and c80 mice, respectively) (Fig 1B). In C57BL/6 c40 mice, the parasites were no longer detected by microscopic examination after 2 days of challenge (Fig 1C), while C57BL/6 c80 mice had limited parasitemia at <0.1% (Fig 1D). Interestingly, CD28KO c40 and c80 mice almost completely controlled the *Pc* re-infection, limiting parasitemia at ~0.1% and ~1%, respectively. In both cases, CD28KO and C57BL/6 negative controls failed to control challenge-induced parasitemia and succumbed (Fig 1C and 1D and data not shown). Furthermore, all the re-infected CD28KO mice (as well as re-infected C57BL/6 mice) survived (data not shown). Our results suggest the existence of an alternative effector mechanism to ensure long-lasting immunity in CD28KO mice.

### Anti-parasite and parasite-unrelated IgM serum levels correlate with delayed parasitemia in CD28KO mice

**Fig 2:**
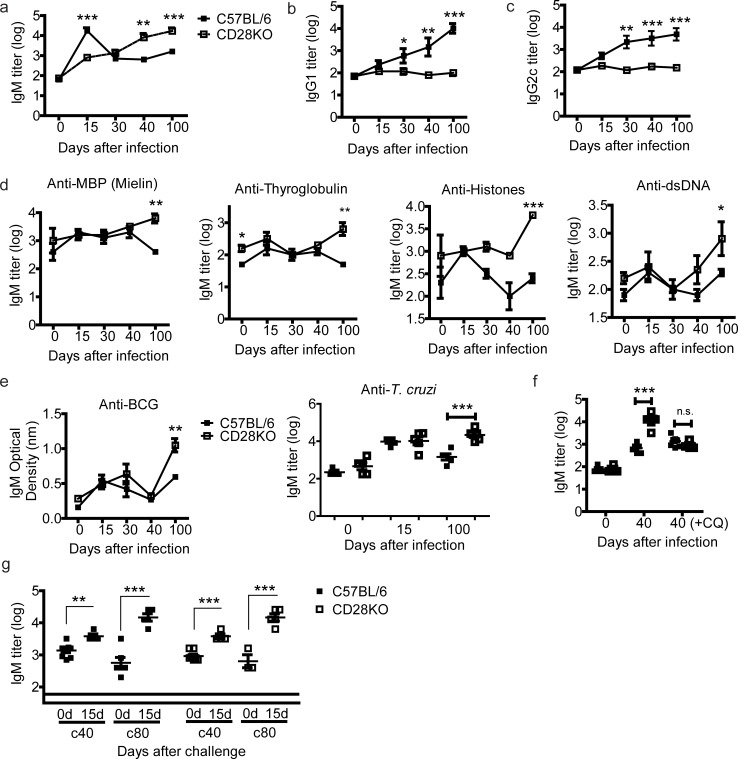
IgM production in C57BL/6 and CD28KO mice during primary and secondary *Pc* infections. (**a-c**) Anti-parasite IgM, IgG1 and IgG2c serum titers during primary infection. (**d-e**) Serum titers of IgM that binds to thyroglobulin (TGB), dsDNA, histone (HST), myelin (MYL), *T*. *cruzi* and BCG at 15 and 100 days after primary infection. (**f**) Anti-parasite IgM serum titers at day 40 p.i. in mice that were or were not treated with chloroquine (CQ). (**g**) Anti-parasite IgM serum titers in c40 and c80 mice. In **a-g**, significant differences (*p<0.05, **p<0.01, ***p<0.001) between the indicated groups are shown. Data from three independent experiments (n = 5–7, mean ± SEM) is shown.

We next investigated the effector mechanisms responsible for *Pc* control in the absence of CD28. First, the anti-parasite serum IgM kinetics were determined in infected C57BL/6 and CD28KO mice. In C57BL/6 mice, anti-parasite IgM peaked at 15 days p.i., and subsequently decreased during chronic infection (Fig 2A). In contrast, infected CD28KO mice had a gradual increase in IgM, achieving maximal levels at 100 days p.i. As previously reported [14], anti-parasite IgG production was completely abrogated in infected CD28KO mice (Fig 2B and 2C). The total IgM kinetics in CD28KO mice (but not C57BL/6) were similar to those observed for the anti-parasite IgM (S1A Fig). Total IgG, however, was not apparently increased in either of the infected mouse groups (S1B and S1C Fig).

The augmented total IgM levels observed led us to investigate whether these antibodies also recognize *Pc*-unrelated antigens, such as autoantigens (e.g., thyroglobulin, histone, dsDNA and myelin) and antigens from other pathogens (e.g., *Trypanosoma cruzi*–*T*. *cruzi* and Bacille Calmette Guerin–BCG). Enhanced IgM titers for all these antigens were observed in CD28KO mice at 100 days p.i. (Fig 2D and 2E). The kinetics of parasite-unrelated IgM followed those of anti-parasite IgM in infected C57BL/6 and CD28KO mice, except for anti-BCG antibodies, which reached higher levels on day 100 p.i. in both mouse groups.

To verify whether persisting infection was responsible for the high IgM serum levels found in the absence of CD28, mice at 30 days p.i. were treated with chloroquine to eliminate residual parasitemia. At day 40 p.i., reductions in the anti-parasite and total IgM levels were observed in cured CD28KO mice (Fig 2F and S1D Fig). Indeed, anti-parasite IgM levels in these mice were similar to those found in C57BL/6 mice (data not shown). Notably, anti-parasite and total IgM levels remained increased in cured CD28KO mice at 80 days p.i. compared with the non-infected controls (Fig 2G and S1E Fig). Secondary infections in cured C57BL/6 and CD28KO mice (at 40 and 80 days p.i.) led to further increases in these levels. C57BL/6 mice also produced high amounts of anti-parasite IgG after re-infection (data not shown).

### Chronically infected CD28KO mice have increased splenic populations of IgM^+^ experienced B cells and IgM^+^ plasmocytes

**Fig 3:**
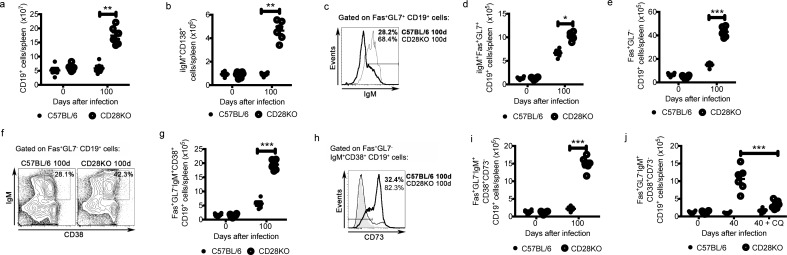
Expansion of the IgM^+^ memory B cell population in the spleens of chronically infected C57BL/6 and CD28KO mice. **(a-i)** Splenic B cells were analyzed in mice on day 100 p.i. and in age-matched controls (0d). **(a)** The CD19^+^ cell numbers per spleen. **(b)** The iIgM^+^CD138^+^ cell numbers per spleen. (**c**) Representative histograms obtained by flow cytometry showing IgM expression in Fas^+^GL7^+^CD19^+^ cells. The IgM^+^ cell percentage data are shown. (**d**) The Fas^+^GL7^+^IgM^+^CD19^+^ cell numbers per spleen. (**e**) The Fas^+^GL7^-^CD19^+^ cell numbers per spleen. (**f**) Representative contour plots obtained by flow cytometry showing IgM and CD38 expression in Fas^+^GL7^-^CD19^+^ cells. The IgM^+^CD38^+^ cell percentage data are shown. (**g**) The Fas^+^GL7^-^IgM^+^CD38^+^CD19^+^ cell numbers per spleen. (**h**) Representative histograms obtained by flow cytometry showing CD73 expression in Fas^+^GL7^-^IgM^+^CD38^+^CD19^+^ cells. The CD73^-^ cell percentage data are shown. (**i**) The Fas^+^GL7^-^IgM^+^CD38^+^CD73^-^CD19^+^ cell numbers per spleen. (**j**) The Fas^+^GL7^-^IgM^+^CD38^+^CD73^-^CD19^+^ cell numbers per spleen at day 40 p.i. in mice that were or were not treated with chloroquine (CQ). In **a-j**, significant differences (*p<0.05, **p<0.01, ***p<0.001) between the indicated groups are shown. Data from three independent experiments (n = 6, mean ± SEM) is shown.

Next, the splenic B cell populations in chronically infected CD28KO mice were characterized. These mice had a dramatic increase in spleen weight and cellularity compared to C57BL/6 mice at 100 days p.i. or to age-matched controls, which was dependent on parasitemia persistence (S2 Fig). The numbers of splenic B (CD19^+^) cells and intracellular (i)IgM^+^CD138^+^ plasmocytes were also significantly higher in infected CD28KO mice (Fig 3A and 3B). Supporting the requirement of CD28 for GC formation [14], the Fas^+^GL7^+^ B cell population was much smaller in infected CD28KO mice than in their C57BL/6 counterparts (S2C and S2D Fig). The few GC-like B cells were IgM^+^ in CD28KO mice (Fig 3C), which were also found in C57BL/6 mice (Fig 3D). Remarkably, on day 100 p.i., CD28KO mice showed a sharp increase in a splenic B cell population that had characteristics of GC-independent IgM^+^ memory B cells [21]. The IgM^+^Fas^+^GL7^-^ B cells (Fig 3E and S2C Fig) expressed IgM and the memory B cell marker, CD38 (Fig 3F and 3G) and were predominantly negative for the ecto-nucleotidase CD73 (Fig 3H and 3I). Residual parasitemia was crucial for the accumulation of this population, as the chloroquine-treated CD28KO mice had lower numbers of these cells (Fig 3J). We herein called this population “IgM^+^ experienced B cells”.

**Fig 4:**
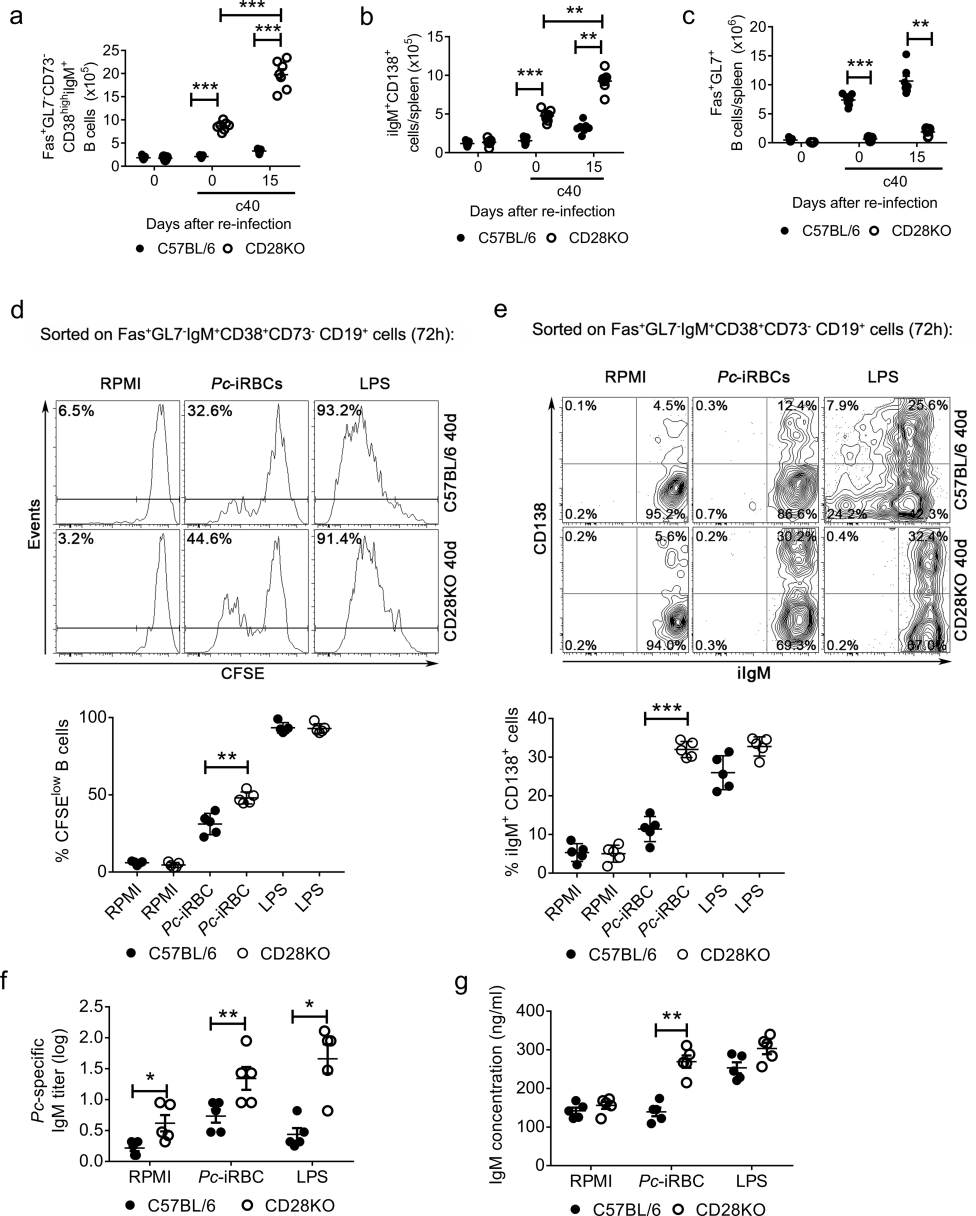
*In vivo* and *in vitro* secondary responses to *Pc*-iRBCs of IgM^+^ memory B cells from cured C57BL/6 and CD28KO mice. (**a**) The Fas^+^GL7^-^IgM^+^CD38^+^CD73^-^CD19^+^ cell numbers per spleen in c40 mice and age-matched controls are shown. (**b**) The IgM^+^CD138^+^ cell numbers per spleen in c40 mice and age-matched controls are shown. (**c**) The Fas^+^GL7^+^CD19^+^ cell numbers per spleen in c40 mice and age-matched controls are shown. (**d-g**) Fas^+^GL7^-^IgM^+^CD38^+^CD73^-^CD19^+^ cells were sorted from infected mice on day 40 p.i., which were previously treated with chloroquine to eliminate reminiscent parasitemia. The cells were stimulated with *Pc*-iRBCs or LPS. (**d**) Representative histograms that were obtained by flow cytometry showing CFSE expression after 72 h of culture. The CFSE^low^ cell percentage data are shown. Compiled percentages of CFSE^low^ cells are shown in the lower panel. (**e**) Representative contour plots obtained by flow cytometry showing CD138 and IgM expression after 72 h of culture. The CD138^+^IgM^+^, CD138^+^IgM^-^, CD138^-^IgM^+^ and CD138^-^IgM^-^ cell percentage data are shown. Compiled percentages of CD138^+^IgM^+^ cells are shown in the lower panel. (**f**) The anti-parasite IgM titers in cell supernatants (from the cultures in **d-e**; 1x10^6^ B cells/well) after 7 days of culture. (**g**) The total IgM concentrations in cell supernatants after 7 days of culture. In **a-g**, the significant differences (*p<0.05, **p<0.01, ***p<0.001) between the indicated groups are shown. Data from three independent experiments (n = 5–6, mean ± SEM) is shown.

We next assessed whether *Pc* parasites could directly induce expansion of IgM^+^ experienced B cells in cured CD28KO mice as well as induce differentiation of these cells for antibody production. The cured CD28KO mice that were challenged with *Pc* parasites displayed increased numbers of Fas^+^GL7^-^IgM^+^CD38^+^CD73^-^ experienced B cells and IgM^+^ plasmocytes (Fig 4A and 4B). Fas^+^GL7^+^ B cells were predominant in cured and *Pc*-challenged C57BL/6 mice (Fig 4C). Sorted IgM^+^ experienced B cells from cured C57BL/6 and CD28KO mice were then stimulated *in vitro* with *Pc*-iRBCs or LPS, which are polyclonal B cell activators [22]. After 72 h of culture, a considerable proportion of IgM^+^ experienced B cells from C57BL/6 and CD28KO mice proliferated and differentiated into iIgM^+^CD138^+^ cells in response to *Pc*-iRBCs, a phenomenon that was also observed following LPS stimulation (Fig 4D and 4E). iIgM^-^ (IgG^+^) plasmocytes, however, were only generated from WT IgM^+^ experienced B cells, but not from CD28KO counterparts (Fig 4E). Anti-parasite and total IgM production levels were also increased in cultured cells that were stimulated with *Pc*-iRBCs (Fig 4F and 4G). Generally, IgM^+^ experienced B cell responses in the CD28KO mice were higher than those in the C57BL/6 mice. Altogether, these results suggest IgM^+^ experienced B cells can differentiate in iIgM^+^ plasmocytes and produce IgM in response to parasite.

### IgM from chronically infected CD28KO mice recognizes mature *Pc*-iRBCs and is effective in parasite control

**Fig 5:**
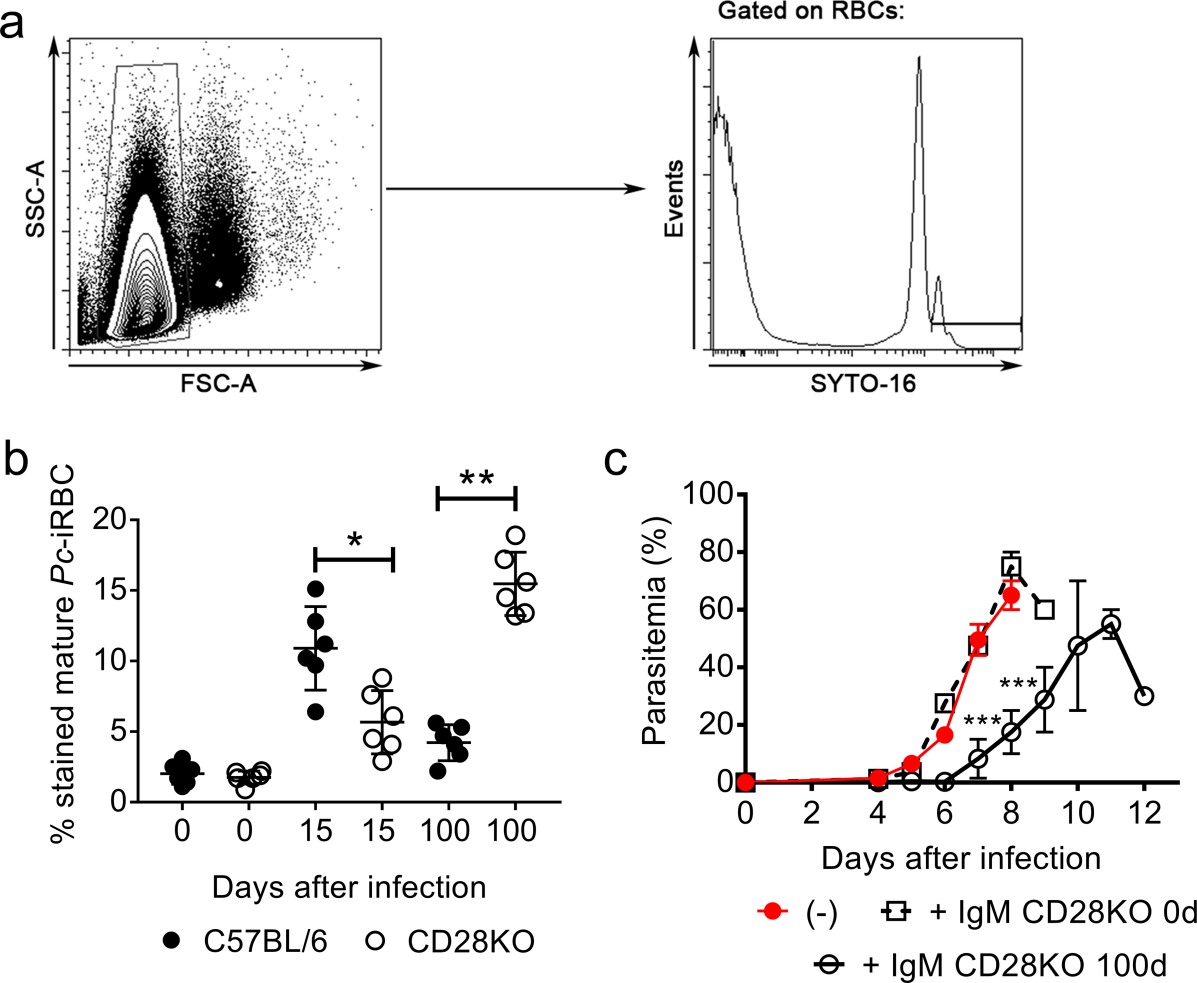
Mature *Pc*-iRBC recognition and *in vivo* parasite control by IgM that was produced by infected C57BL/6 and CD28KO mice. **(a-c)** IgM was purified from serum samples of mice on days 15 and 100 p.i. and from age-matched controls (0d). (**a**) Representative histogram obtained by flow cytometry showing gating of mature *Pc*-iRBCs (SYTO 16^high^ RBCs) from RAGKO mice. (**b**) Percentages of IgM^+^ mature Pc-iRBCs are shown. (**c**) Parasitemia curves in RAGKO mice that were previously i.v. treated with purified IgM and then i.p. infected with 1 x 10^6^ *Pc*-iRBCs. In **b-c,** Significant differences (*p<0.05, **p<0.01, ***p<0.001) are shown. In **b-c**, data from three independent experiments (n = 6–7, mean ± SEM) is shown.

We next evaluated whether IgM from CD28KO mice recognized *Pc*-iRBCs. Antibody-free *Pc*-iRBCs (isolated from infected RAGKO mice) were incubated with IgM from chronically infected C57BL/6 or CD28KO mice. IgM from 15 day-infected C57BL/6 and from 100 day-infected CD28KO mice recognized higher proportions of mature *Pc*-iRBCs compared to IgM from naïve mice (Fig 5A). Furthermore, IgM from 100 day-infected CD28KO mice transferred protection to RAGKO mice (Fig 5B). A delay in parasitemia of several days was observed after this treatment, while IgM from non-infected CD28KO mice was not protective.

**Fig 6:**
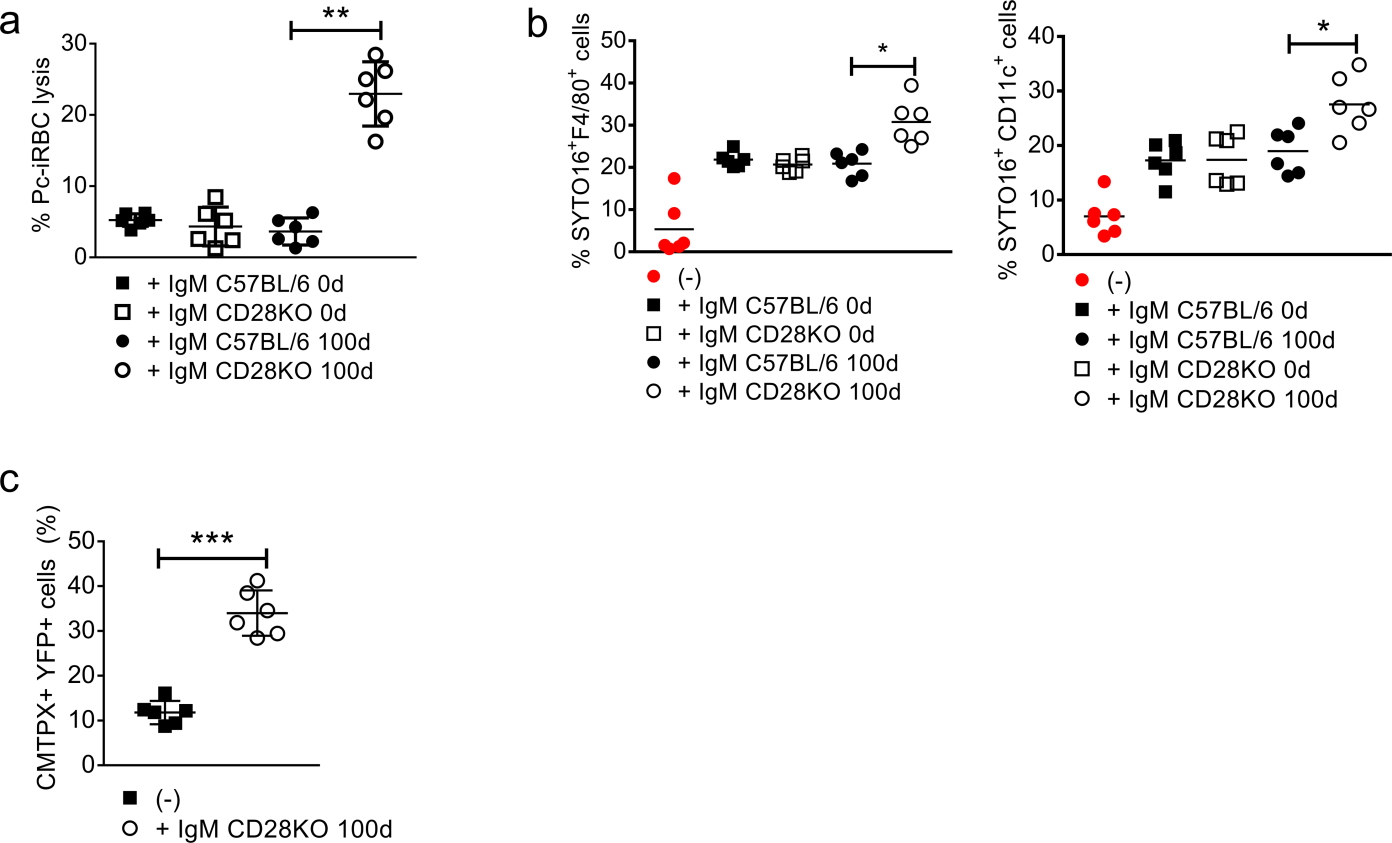
Lysis and phagocytosis of *Pc*-iRBCs that was mediated by IgM produced from chronically infected C57BL/6 and CD28KO mice. **(a-c)** IgM was purified from serum samples of mice on day 100 p.i. and age-matched controls (0d). **(a)** Lysis of mature *Pc*-iRBCs that was mediated by purified IgM in the presence of complement components (RAGKO mouse serum). The iRBC lysis percentages were determined in relation to negative (PBS) and positive (H_2_O) controls. **(b)** *In vitro* uptake by splenic F4/80^+^ macrophages and dendritic cells of mature iRBCs, which were or were not previously incubated with purified IgM from chronically infected CD28KO mice. SYTO 16-labeled *Pc*-iRBCs from RAGKO mice were incubated with purified IgM for 30 min and then cultured with phagocyte-enriched splenocytes for 20 min. The SYTO 16^+^F4/80^+^ cell and SYTO 16^+^CD11c^+^ cell percentage data are shown, which was obtained by flow cytometry. **(c)** *In vivo* uptake by splenic red pulp dendritic cells of mature iRBCs, which were or were not previously incubated with purified IgM. The CMTPX-labelled iRBCs from the RAGKO mice was incubated with purified IgM and inoculated i.v. into CD11c-YFP mice. The scale bars represent 50 μm. The CMTPX^+^YFP^+^ cell percentage data in the YFP^+^ cell population is shown. In **a-c**, significant differences (*p<0.05, **p<0.01, ***p<0.001) between the experimental groups are shown. Data from three independent experiments (n = 6, mean ± SEM) is shown.

We also assessed the functionality of IgM from 100 day-infected CD28KO mice. These antibodies significantly enhanced complement-mediated *Pc*-iRBC lysis, while IgM from 100 day-infected C57BL/6 mice or non-infected controls were ineffective (Fig 6A). IgM from 100 day-infected CD28KO mice also increased the *in vitro* uptake of SYTO 16-labeled mature *Pc*-iRBCs by splenic CD11c^+^ (dendritic cells) and F4/80^+^ cells (macrophages) (Fig 6B). Moreover, pre-incubation with these antibodies enhanced the *in vivo* uptake of mature *Pc*-iRBCs by splenic dendritic cells. This was demonstrated by inoculating cell tracker orange (CMTPX)-labelled mature *Pc*-iRBCs, which was or was not pre-incubated with IgM from 100 day-infected CD28KO mice, into CD11c.YFP mice. Intravital imaging revealed enhanced 3D co-localization of mature *Pc*-iRBCs with CD11c^+^YFP^+^ cells inside the splenic red pulp in the presence of antibodies (Fig 6C). Overall, these results suggest IgM produced in response to *Pc* infection induces multiple mechanisms that can recognize and act on *Plasmodium* parasites.

## Discussion

In this study, we evaluated the role of IgM produced in response to experimental malaria using chronically infected CD28KO mice as a model, which showed increased parasitemia due to a defect in developing classic memory T and B cells [14]. Interestingly, *Pc*-infected CD28KO mice acquired long-lasting non-sterile immunity and survived a lethal parasite challenge. The protective immunity in CD28KO mice is associated with a progressive increase in anti-parasite and total IgM and, notably, these antibodies recognize *Pc*-iRBCs and promote parasite clearance. Additionally, the analysis of chronically infected CD28KO mice allowed us to characterize a population of Fas^+^GL7^-^IgM^+^CD38^+^CD73^-^ B cells, which was also present in lower numbers in chronically infected C57BL/6 mice. These IgM^+^ B cells differentiated into IgM^+^ plasmocytes and produced anti-parasite IgM in response to *Pc*-iRBCs, in a T cell-independent manner, suggesting these cells could be a source of IgM-producing cells in our model. As showed in Fig 2G, there is an increase in production of IgM in response to challenge in cured mice, indicating a “memory-like” response for B cells in both C57BL/6 and CD28KO mice. Although this could indicate the IgM^+^ B cells found in this context to be memory cells, the presence of patent blood parasitemia was fundamental for their accumulation, which could indicate these cells as being “constantly activated/effector” B cells rather than bona-fide memory. Hence, this population can be better defined as “IgM^+^ experienced B cells”. We also found that the vast majority of the few cells with a GC phenotype in CD28KO mice are IgM^+^. Although this could indicate the generation of functional germinal centers in the absence of CD28 co-stimulation generate preferentially unswitched cells, the low numbers of these cells make this assumption unlikely. Overall, this study helps to understand the role of IgM in chronic malaria.

The role of IgM in protective immunity against *Plasmodium* infection is still controversial, where both a positive role via production of neutralizing IgM [23] and reduced effector function associated with IgM were reported [24]. Our results indicate that IgM plays an important protective role in situations where conventional acquired immunity is not optimal, such as immunodeficiency and prolonged chronic parasitemia in face of impaired classical immunological memory. The function of these cells in C57BL/6 mice is unclear. The high levels of IgG specific antibodies, together with higher levels of CD4+ T cell memory, might mask some of the protective functions that could be attributed to IgM (and IgM^+^ experienced B cells). Moreover, the presence of patent chronic parasitemia favors the expansion of these cells (Fig 3), which does not happen in C57BL/6 mice. Nevertheless, IgM isolated from d100 C57BL/6 mice can lead to phagocytosis and complement induced lysis, albeit not in the same extent to the observed in CD28KO mice (Fig 6). This makes it possible that IgM from B6 mice is indeed functional. This finding supports the speculation that IgM^+^ memory B cells are remnants of primitive immune system that persisted throughout evolution [25] and thus act as a backup protective mechanism upon infection. Like innate B cells [26], these cells could have been evolutionarily selected to produce germ-line gene encoded polyreactive antibodies that bind to multiple conserved microbial molecules and damage-associated self-components. Reinforcing this idea, we showed that IgM produced by chronically infected CD28KO mice (in reaction to the presence of patent blood *Pc* infection) recognized the parasite as well as autoantigens and antigens from other pathogens. Uninfected CD28KO mice were not producing these antibodies–as we stated above, this is believed to be mainly a result of polyclonal activation in response to patent blood-stage infection. Alternatively, the prevalence and accumulation of IgM did not prevent parasite persistence in CD28KO mice, which could indicate that the parasite evolved mechanisms that can subvert the immune response into a less effective type in certain circumstances. The notion of malaria-induced autoantibodies is not new; in humans, autoantibodies are produced during malaria infection [27]. A logical prediction would be that the recognition of auto-antigens by malaria-induced IgM could induce autoimmunity at some extent in chronically infected mice. We did not observe any effect on our experimental mice. Perhaps at later time points (which we did not explore) we could have observed such consequences.

The protective role of IgM was suggested by our data showing that IgM from chronically infected CD28KO mice was more efficient in limiting parasite replication *in vivo* than IgM from naïve CD28KO mice. IgM from chronically infected CD28KO mice efficiently recognized and opsonized *Pc*-iRBCs, which led to phagocytosis by splenic phagocytes, as well as inducing complement-dependent *Pc*-iRBCs lysis. These antibodies may target *Pc*-iRBCs by binding to parasite antigens that are expressed on the erythrocyte surface or damage-associated self-molecules, such as Band 3 clusters [28][29]. In support of our data, anti-parasite IgM has been previously shown to protect against malaria in mouse models [30][31]. In humans, binding of non-immune or ‘natural’ IgM to the *P*. *falciparum*-iRBC surface is supposedly due to the interaction of a subset of the parasite variant surface antigen, *P*. *falciparum* erythrocyte membrane protein 1 (PfEMP1), with the Fc regions of IgM [32]. This interaction is often interpreted as deleterious to the host as it leads to the formation of rosettes that induce immunopathology [33], and it contributes to immune system evasion by masking of protective IgG epitopes [34]. However, the positive correlation of IgM response to parasite antigens with resistance to *P*. *falciparum* infection in African ethnic groups indicates a protective role of these antibodies [35].

Altogether, our study reveals a protective role for IgM in experimental malaria. It also shows that increased IgM production occurs when infection develops in the absence of classic memory T and B cells. Moreover, IgM production is dependent upon parasite persistence. These results may help to explain why IgM-producing B cells are expanded in malaria patients living in endemic areas [35]. The rapid decline of anti-parasite IgG serum levels after interruption of parasite exposure suggests classical humoral memory is functionally impaired in malaria patients [35, 36]. As a counterpart, IgM^+^ memory B cells might arise as a primitive, innate-like defence mechanism against infection.

## Materials and methods

### Mice, parasites, infection and chloroquine treatment

Six- to eight-week-old C57BL/6, CD28KO, RAGKO and CD11c.YFP (all in a C57BL/6 background) female mice (Jackson Laboratory, USA) were bred under specific pathogen-free conditions at the Isogenic Mouse Facility (ICB–USP). *Pc* parasites (AS strain) were maintained as described [37]. Mice were inoculated intraperitoneally (i.p.) with 1 x 10^6^ *Pc*-iRBCs for primary infections and 1 x 10^8^ *Pc*-iRBCs for secondary infections and intravenously (i.v.) with 1 x 10^8^ *Pc*-iRBCs to assess *in vivo* phagocytosis. For complete parasite elimination, mice were treated i.p. with 10 mg/kg body weight/day of chloroquine (Sigma-Aldrich, USA) over 8 consecutive days. Parasitemia was determined by microscopic examination of Giemsa-stained blood smears. Mice were sacrificed using a CO_2_ chamber.

### Ethics statement

All procedures were in accordance with the national regulations of ethical guidelines for mouse experimentation and welfare of the Conselho Nacional de Saúde and Colégio Brasileiro em Experimentação Animal (COBEA)—Brazil, and the protocols were approved by the Health Animal Committee (Comissão de Ética no Uso de Animais–CEUA–ICB—USP), with permit numbers 0174/2011 and 0036/2007.

### Spleen cell suspensions

Spleen cells were washed and maintained in RPMI 1640 that was supplemented with penicillin (100 U/ml), streptomycin (100 μg/ml), 2-mercaptoethanol (50 μM), L-glutamine (2 mM), sodium pyruvate (1 mM) and 10% heat-inactivated foetal calf serum. All supplements were purchased from Life Technologies (USA). To obtain phagocyte-enriched splenocyte suspensions, spleen cells were treated with type II collagenase (Invitrogen, USA) at a final concentration of 0.5 mg/ml for 40 min at 37°C in a 5% CO_2_ atmosphere.

### Spleen cell phenotyping

Splenocytes were stained with fluorophore-labelled mAbs to CD19 (1D3), CD38 (90), Fas (CD95, Jo2), GL7 (GL7), CD73 (eBioTy/11.8), CD138 (Syndecan, 281–2) and IgM^b^ (AF6-78). For the iIgM staining, spleen cells were cultured with GolgiStop for 6 h at 37°C in a 5% CO_2_ atmosphere according to the manufacturer’s instructions (BD Biosciences). After surface phenotyping, the cells were fixed with Cytofix/Cytopern buffer and stained with PE-labelled mAb to IgM. All reagents were obtained from BD Biosciences (USA), except for CD73, F4/80 and CD11c, which were purchased from eBioscience (USA). Cells were analysed by flow cytometry with the FACSCalibur or FACSCanto devices (BD Biosciences) and the FlowJo 9.5.3 software (Tree Star Inc., USA).

### *In vitro* stimulation of IgM^+^ memory B cells

Fas^+^GL7^-^IgM^+^CD38^+^CD73^-^CD19^+^ cells were sorted with a FACSAria device (BD Biosciences), and then they were stained with 5 μM carboxyfluorescein succinimidyl ester (CFSE, Life Technologies) in PBS with 0.1% bovine serum albumin (BSA, Sigma-Aldrich) for 20 min at 37°C. The cells were then stimulated with *Pc*-iRBCs (1 B cell: 3 *Pc*-iRBCs) or LPS (10 μg/ml, from *Escherichia coli* 0111:B4, Sigma-Aldrich). Proliferation was evaluated after 72 h of culture at 37°C in a 5% CO_2_ atmosphere, whereas supernatants were collected at 7 days to evaluate the total and anti-parasite IgM levels.

### ELISA for antibody quantification

Ninety-six-well, flat-bottom microtest plates (Costar, USA) were coated overnight (4°C) with an intraerythrocytic *Pc* extract (10 μg/ml) [38], *Trypanosoma cruzi* (Y strain) extract (10 μg/ml) [39], BCG extract (10 μg/ml), myelin (10 μg/ml; Invitrogen), thyroglobulin (10 μg/ml; Invitrogen), histone (10 μg/ml; Invitrogen), dsDNA (10 μg/ml; Invitrogen) or purified sheep anti-mouse total Ig antibody (10 μg/ml). The plates were saturated with 1% BSA for 2 h. After washing, 50 μl of mouse serum samples (diluted from 1:10 to 1:128,000) were added and left for 2 h at room temperature (RT). Total antibody concentrations were determined using IgG1, IgG2c or IgM standards. The assays were developed by adding goat anti-mouse IgG1, IgG2c or IgM peroxidase-conjugated antibodies for 1 h, followed by 100 μl of tetra-methyl-benzidine (TMB, Life Technologies). All antibodies were obtained from Southern Biotechnology Associates (USA). Absorbance was measured at 650 nm with a Spectra Max 190 spectrophotometer. Total antibody levels were presented as concentration (μg/ml), while specific antibody levels were presented as titers. All plates had standard curves, as well as blank wells which consisted of non-coated wells.

### IgM purification

Mouse serum IgM was purified by affinity chromatography in a G-Sepharose protein column (GE Healthcare, USA). Sepharose-bound IgM was eluted with 0.01 M sodium phosphate buffer (pH 7.0) and monitored by absorbance readings at 280 nm. The eluted samples were dialyzed in PBS, concentrated and stored at -20° C. The purity of IgM after isolation was >98% in all samples. Equilibration was performed with 20 mM sodium phosphate, 800 mM ammonium sulfate, pH 7.5.

### IgM binding to mature *Pc*-iRBCs

Antibody-free *Pc*-iRBCs were obtained from the blood of infected RAGKO mice during a period of the circadian parasite cycle in which mature stages predominate (>95% late trophozoites/schizonts). For our convenience, the mice were placed in an animal room with an inverted light/dark cycle for at least 15 days before infection. Blood cells (5 x 10^7^) were incubated with purified IgM (at a final concentration of 20 ηg/ml) for 45 min at 37°C. The cells were stained with PE-labelled anti-IgM^b^ mAb and 5 μM SYTO 16 (Invitrogen) and were then analysed by flow cytometry.

### Isolation and staining of antibody-free mature *Pc*-iRBCs

Antibody-free mature *Pc*-iRBCs (>95% late trophozoites/schizonts) were obtained from the blood of infected RAGKO mice. Blood samples (500 μl) were resuspended in 1 ml PBS, pipetted over 5 ml of 74% Percoll (GE Healthcare) and centrifuged (2,500 x g, acceleration/break 5/0) for 30 min at RT. The top cell layers were collected and washed with supplemented RPMI 1640 medium. Purified mature *Pc*-iRBCs (>95% purity) were stained with 5 μM SYTO 16 (Invitrogen) or CMTPX (Life Technologies) following the manufacturer’s instructions.

### Adoptive transfer of purified IgM

Purified IgM samples (500 μl at 20 ηg/ml) were inoculated i.v. into RAGKO mice after 24 h of infection with 1 x 10^6^ *Pc*-iRBCs, and parasitemia was monitored as described above.

### IgM-mediated lysis of mature *Pc*-iRBCs

The ability of IgM to induce complement-mediated lysis of *Pc*-iRBCs was evaluated with an adaptation of the haemolysis assay [40]. In brief, 5 x 10^7^ antibody-free mature *Pc*-iRBCs were pre-incubated with purified IgM as described above and then maintained for 45 min at 37°C with 50 μl of RAGKO mouse serum, as an antibody-free source of complement components. The background and total cell lysis levels were evaluated by incubation of *Pc*-iRBCs with PBS (background) and H_2_O (total cell lysis). The absorbance of the supernatant was measured at 414 nm with a Spectra Max 190 spectrophotometer and expressed as lysis percentage.

### *In vitro* phagocytosis of mature *Pc*-iRBCs

Mature *Pc*-iRBCs were pre-incubated with purified IgM and stained with SYTO 16, as described above. These cells (3 x 10^6^) were then cultured with phagocyte-enriched splenocytes (1 x 10^6^) for 20 min at 37°C in a 5% CO_2_ atmosphere and analysed by flow cytometry.

### *In vivo* phagocytosis of mature *Pc*-iRBCs

Phagocytosis of *Pc*-iRBCs by splenic dendritic cells was previously reported [41]. CD11c.YFP mice were inoculated i.v. with CMTPX-stained mature *Pc*-iRBCs (1 x 10^8^), which were or were not pre-incubated with purified IgM as described above. After 30 min, the mice were deeply i.p. anesthetized with 55 ng/g/body weight of ketamine (Imalgene 1000, Merial, USA) and 0.85 ng/g/body weight of xylazine (Rompun 2%, Bayer, Germany). The spleens were externalized, and live imaging was conducted with a LSM 780-NLO confocal microscope (Zeiss, Germany). The data were processed with the ZEN 2012 lite software (Zeiss). The 28 μm Z-sections with 4 μm Z-increments were acquired for 30 min. An Imaris X64 7.0.0 (Andor Technology) was used to edit images and to determine the CMTPX^+^CD11c^+^ cell percentages.

### Statistical analysis

Statistical analyses were performed with the GraphPad Prism 5 software (GraphPad, USA), and the differences between the groups were considered significant when *p* < 0.05 (5%). The simultaneous effects of two factors were analysed with the two-way ANOVA and the Bonferroni post-hoc test. The one-way ANOVA and the Tukey post-hoc test was used to assess the effects of only one factor. All samples were evaluated by the Kolmogorov-Smirnov test to assess Gaussian distribution of samples.

## Supporting information

S1 Fig Total IgM and IgG production in C57BL/6 and CD28KO mice during primary and secondary *Pc* infections. (**a-c**) Total IgM, IgG1 and IgG2c serum concentrations during primary infection. (**d**) Total IgM serum concentrations at day 40 p.i., in mice that were or were not treated with chloroquine (CQ). (**e**) Total IgM serum concentrations in c40 and c80 mice. In **a-e**, significant differences (p < 0.05) between the indicated groups are designated by *. One representative experiment out of three (n = 3–7, means ± SEM) is shown.
(TIF)

S2 Fig Spleen weight and cell numbers in chronic and cured C57BL/6 and CD28KO mice. **(a-d)** Mice on day 30 p.i., were treated or not with chloroquine (+CQ) to eliminate reminiscent parasitemia or not (-CQ) and analyzed on day 100 p.i. (**a**) Data showing spleen weights. (**b**) Data showing total numbers of spleen cells. (**c**) Representative contour plots obtained by flow cytometry showing Fas and GL7 expression in CD19^+^ cells. The Fas^+^GL7^+^ and Fas^+^GL7^-^ cell percentage data are shown. (**d**) The Fas^+^GL7^+^CD19^+^ cell numbers per spleen. In **a-d**, significant differences (*p<0.05, **p<0.01, ***p<0.001) between all experimental groups (C57BL/6 and CD28KO) are shown. Data from three independent experiments (n = 6–7, means ± SEM) is shown.
(PDF)

S1 Dataset Full list of individual values for all experiments listed on this manuscript. (XLSX)

## References

[1] Cohen S, McGregor I, Carrington S. Gamma-globulin and acquired immunity to human malaria. Nature. 1961;192:733–7. 13880318 10.1038/192733a0

[2] Chan J, Howell KB, Reiling L, Ataide R, Mackintosh CL, Fowkes FJI, et al Targets of antibodies against Plasmodium falciparum–infected erythrocytes in malaria immunity. J Clin Invest. 2012;122(9). 10.1172/JCI62182 PMC3428085 22850879

[3] Langhorne J, Gillard S, Simon B, Slade S, Eichmann K. Frequencies of CD4+ T cells reactive with Plasmodium chabaudi chabaudi: distinct response kinetics for cells with Th1 and Th2 characteristics during infection. Int Immunol. 1989;1:416–24. 2535135 10.1093/intimm/1.4.416

[4] Muxel SM, Freitas do Rosario AP, Zago CA, Castillo-Mendez SI, Sardinha LR, Rodriguez-Malaga SM, et al The Spleen CD4 + T Cell Response to Blood-Stage Plasmodium chabaudi Malaria Develops in Two Phases Characterized by Different Properties. PLoS One. 2011;6(7):1–12. 10.1371/journal.pone.0022434 PMC3141041 21814579

[5] Stephens R, Culleton RL, Lamb TJ. The contribution of Plasmodium chabaudi to our understanding of malaria. Trends Parasitol [Internet]. 2015;28(2):74–83. Available from: 10.1016/j.pt.2011.10.006 PMC4040349 22100995

[6] Stevenson M, Tam M. Differential induction of helper T cell subsets during blood-stage Plasmodium chabaudi AS infection in resistant and susceptible mice. Clin Exp Immunol. 1993;92:77–83. 8096804 10.1111/j.1365-2249.1993.tb05951.x PMC1554870

[7] D’Imperio Lima M, Alvarez J, Furtado G, Kipnis T, Coutinho A, Minoprio P. Ig-Isotype Patterns of Primary and Secondary B Cell Responses to Plasmodium Chabaudi Chabaudi Correlate with IFN- and IL-4 Cytokine Production and with CD45RB Expression by CD4 ‡ Spleen. Scand J Immunol. 1996;43:263–70. 8602459 10.1046/j.1365-3083.1996.d01-35.x

[8] Lugaajju A, Reddy SB, Wahlgren M, Kironde F, Persson KEM. Development of Plasmodium falciparum specific naïve, atypical, memory and plasma B cells during infancy and in adults in an endemic area. Malar J. 2017;1–11. 10.1186/s12936-016-1650-6 28109284 PMC5251336

[9] Akhouri RR, Goel S, Furusho H, Skoglund U, Wahlgren M. Architecture of Human IgM in Complex with Article Architecture of Human IgM in Complex with P. falciparum Erythrocyte Membrane Protein 1. Cell Rep [Internet]. 2016;14(4):723–36. Available from: 10.1016/j.celrep.2015.12.067 26776517

[10] Pleass RJ, Moore SC, Stevenson L, Hviid L. Immunoglobulin M: Restrainer of In fl ammation and Mediator of Immune Evasion by Plasmodium falciparum Malaria. Trends Parasitol [Internet]. 2016;32(2):108–19. Available from: 10.1016/j.pt.2015.09.007 26597020

[11] Krishnamurty Akshay T Thouvenel CD, Portugal S, Keitany GJ, Kim KS, Holder A, Crompton PD, et al Somatically Hypermutated Plasmodium- Specific IgM + Memory B Cells Are Rapid, Plastic, Early Responders upon Malaria Rechallenge Article Somatically Hypermutated Plasmodium- Specific IgM + Memory B Cells Are Rapid, Plastic, Early Responders upon Malar. Immunity [Internet]. 2016;45(2):402–14. Available from: 10.1016/j.immuni.2016.06.014 27473412 PMC5118370

[12] Harding F, McArthur J, Gross J, Raulet D, Allison J. CD28-mediated signaling co-stimulates murine T cells and prevents induction of anergy in T-cell clones. Nature. 1992;356:607–9. 10.1038/356607a0 1313950

[13] Ferguson S, Han S, Kelsoe G, Thompson C. CD28 is required for germinal center formation. J Immunol. 1996;156:4576–81. 8648099

[14] Elias RM, Sardinha LR, Bastos KRB, Zago CA, Freitas do Rosario AP, Alvarez J, et al Role of CD28 in Polyclonal and Specific T and B Cell Responses Required for Protection against Blood Stage Malaria. J Immunol. 2005;174:790–9. 15634900 10.4049/jimmunol.174.2.790

[15] Atkinson JP, Frank MM. the In Vivo Effects of Antibody INTERACTION OF IgM ANTIBODY AND COMPLEMENT IN THE IMMUNE CLEARANCE AND DESTRUCTION OF ERYTHROCYTES IN MAN. J Clin Invest. 1974;54(January):339–48. 4847248 10.1172/JCI107769 PMC301561

[16] Fossati-jimack BL, Reininger L, Chicheportiche Y, Clynes R, Ravetch J V, Honjo T, et al High Pathogenic Potential of Low-Affinity Autoantibodies in Experimental Autoimmune Hemolytic Anemia. J Exp Med. 1999;190(11):1689–96. 10587359 10.1084/jem.190.11.1689 PMC2195740

[17] Ogden C, Kowalewski R, Peng Y, Montenegro V, Elkon K. IGM is required for efficient complement mediated phagocytosis of apoptotic cells in vivo. Autoimmunity. 2005;38:259–64. 16206508 10.1080/08916930500124452

[18] Kubagawa H, Oka S, Kubagawa Y, Torii I, Takayama E, Kang D, et al Identity of the elusive IgM Fc receptor (Fc u R) in humans. J Exp Med. 2009;206(12):2779–93. 10.1084/jem.20091107 19858324 PMC2806608

[19] Snapper CM. Mechanisms underlying in vivo polysaccharide-specific immunoglobulin responses to intact extracellular bacteria. Ann N Y Acad Sci. 2012;1253:92–101. 10.1111/j.1749-6632.2011.06329.x 22288494

[20] Bruyn G, Zegers B, van Furth R. Mechanisms of host defense against infection with Streptococcus pneumoniae. Clin Infect Dis. 1992;14:251–62. 1571441 10.1093/clinids/14.1.251

[21] Taylor JJ, Pape KA, Jenkins MK. A germinal center–independent pathway generates unswitched memory B cells early in the primary response. J Exp Med. 2012;209(3):597–606. 10.1084/jem.20111696 22370719 PMC3302224

[22] Andersson J, Sjoberg O, Moller G. Induction of immunoglobulin and antibody synthesis in vitro by lipopolysaccharides. Eur J Immunol. 1972;2:349–53. 10.1002/eji.1830020410 4563347

[23] Muellenbeck MF, Ueberheide B, Amulic B, Epp A, Fenyo D, Busse CE, et al Atypical and classical memory B cells produce Plasmodium falciparum neutralizing antibodies. J Exp Med. 2013;210(2):389–99. 10.1084/jem.20121970 23319701 PMC3570107

[24] Portugal S, Tipton CM, Sohn H, Kone Y, Wang J, Li S, et al Malaria-associated atypical memory B cells exhibit markedly reduced B cell receptor signaling and effector function. Elife. 2015;4:1–21. 10.7554/eLife.07218 PMC4444601 25955968

[25] Capolunghi F, Rosado MM, Sinibaldi M, Aranburu A, Carsetti R. Why do we need IgM memory B cells? Immunol Lett [Internet]. 2013;152(2):114–20. Available from: 10.1016/j.imlet.2013.04.007 23660557

[26] Bendelac A, Bonneville M, Kearney JF. AUTOREACTIVITY BY DESIGN: INNATE B AND T LYMPHOCYTES. Nat Rev Immunol. 2001;1(December):177–86. 11905826 10.1038/35105052

[27] Gomes LR, Martins YC, Ferreira-da-cruz MF, Daniel-Ribeiro CT. Autoimmunity, phospholipid-reacting antibodies and malaria immunity. Lupus. 2014;23:1295–8. 10.1177/0961203314546021 25228731

[28] Pantaleo A, Giribaldi G, Mannu F, Arese P, Turrini F. Naturally occurring anti-band 3 antibodies and red blood cell removal under physiological and pathological conditions. Autoimmun Rev. 2008;7:457–62. 10.1016/j.autrev.2008.03.017 18558362

[29] Wolofsky KT, Ayi K, Branch DR, Hult AK, Olsson ML, Liles C, et al ABO Blood Groups Influence Macrophage-mediated Phagocytosis of Plasmodium falciparum-infected Erythrocytes. PLoS Pathog. 2012;8(10):e1002942 10.1371/journal.ppat.1002942 23071435 PMC3469569

[30] Harte P, Cooke A, Playfair J. Specific monoclonal IgM is a potent adjuvant in murine malaria vaccination. Nature. 1983;302:256–8. 6835362 10.1038/302256a0

[31] Couper KN, Phillips RS, Brombacher F, Alexander J. Parasite-specific IgM plays a significant role in the protective immune response to asexual erythrocytic stage Plasmodium chabaudi AS infection *. Parasite Immunol. 2005;2807(May):171–80. 10.1111/j.1365-3024.2005.00760.x 15987340

[32] Semblat J, Raza A, Kyes SA, Rowe JA. Identification of Plasmodium falciparum var1CSA and var2CSA domains that bind IgM natural antibodies. Mol Biochem Parasitol. 2006;146:192–7. 10.1016/j.molbiopara.2005.12.007 16442168 PMC2869447

[33] Stevenson L, Huda P, Jeppesen A, Laursen E, Rowe JA, Craig A, et al Investigating the function of F c -specific binding of IgM to Plasmodium falciparum erythrocyte membrane protein 1 mediating erythrocyte rosetting. Cell Microbiol. 2015;17(January):819–31. 25482886 10.1111/cmi.12403 PMC4737123

[34] Barfod L, Dalgaard MB, Pleman ST, Ofori MF, Pleass RJ, Hviid L. Evasion of immunity to Plasmodium falciparum malaria by IgM masking of protective IgG epitopes in infected. Proc Natl Acad Sci. 2011;(18):1–6. 10.1073/pnas.1103708108 PMC3145728 21746929

[35] Arama C, Skinner J, Doumtabe D, Tran TM, Jain A, Traore B, et al Genetic Resistance to Malaria Is Associated With Greater Enhancement of Immunoglobulin (Ig) M Than IgG Responses to a Broad Array of Plasmodium falciparum Antigens. Open Forum Infect Dis. 2018;(February):1–9. 10.1093/ofid/ofv118 PMC4564391 26361633

[36] Langhorne J, Ndungu FM, Sponaas A, Marsh K. Immunity to malaria: more questions than answers. Nat Immunol. 2008;9(7):725–32. 10.1038/ni.f.205 18563083

[37] Podoba JE, Stevenson MM. CD4 + and CD8 + T Lymphocytes Both Contribute to Acquired Immunity to Blood-Stage Plasmodium chabaudi AS. Infect Immun. 1991;59(1):51–8. 1898902 10.1128/iai.59.1.51-58.1991 PMC257704

[38] Lima M, Bandeira A, Falanga P, Freitas A, Kipnis T, Silva L da, et al Clonal analysis of B lymphocyte responses to Plasmodium chabaudi infection of normal and immunoprotected mice. Int Immunol. 1991;3:1207–16. 1777417 10.1093/intimm/3.12.1207

[39] Marinho CRF, D Imperio Lima MR, Grisotto MG, Alvarez JM. Influence of Acute-Phase Parasite Load on Pathology, Parasitism, and Activation of the Immune System at the Late Chronic Phase of Chagas ‘ Disease. Infect Immun. 1999;67(1):308–18. 9864231 10.1128/iai.67.1.308-318.1999 PMC96312

[40] Tambourgi D V, Fernandes Pedrosa MF, Goncalves de Andrade RM, Billington SJ, Griffiths M, van der Berg CW. Sphingomyelinases D induce direct association of C1q to the erythrocyte membrane causing complement mediated autologous haemolysis. Mol Immunolgy. 2007;44:576–82. 10.1016/j.molimm.2006.02.002 16540172

[41] Borges da Silva H, Fonseca R, Cassado AA, Machado de Salles É, de Menezes M, Langhorne J, et al In Vivo Approaches Reveal a Key Role for DCs in CD4 + T Cell Activation and Parasite Clearance during the Acute Phase of Experimental Blood-Stage Malaria. PLoS Pathog. 2015;11:1–24. 10.1371/journal.ppat.1004598 PMC4450059 25658925

