# Correction: CD28 deficiency leads to accumulation of germinal-center independent IgM^+^ experienced B cells and to production of protective IgM during experimental malaria

**DOI:** 10.1371/journal.pone.0211506

**Published:** 2019-03-25

**Authors:** Henrique Borges da Silva, Érika Machado de Salles, Eliana Lima Faquim-Mauro, Luiz Roberto Sardinha, José Maria Álvarez, Maria Regina D’Império Lima

The third author’s name is spelled incorrectly. The correct name is: Eliana Lima Faquim-Mauro. The correct citation is: Borges da Silva H, Machado de Salles É, Faquim-Mauro EL, Sardinha LR, Álvarez JM, D’Império Lima MR (2018) CD28 deficiency leads to accumulation of germinal-center independent IgM^+^ experienced B cells and to production of protective IgM during experimental malaria. PLoS ONE 13(8): e0202522. https://doi.org/10.1371/journal.pone.0202522
