# Supplementary figures and images for: CD28 deficiency leads to accumulation of germinal-center independent IgM+ experienced B cells and to production of protective IgM during experimental malaria
Source: PLoS One. 2018 Aug 27;13(8):e0202522. doi: 10.1371/journal.pone.0202522 (PMC6110469; doi:10.1371/journal.pone.0202522)

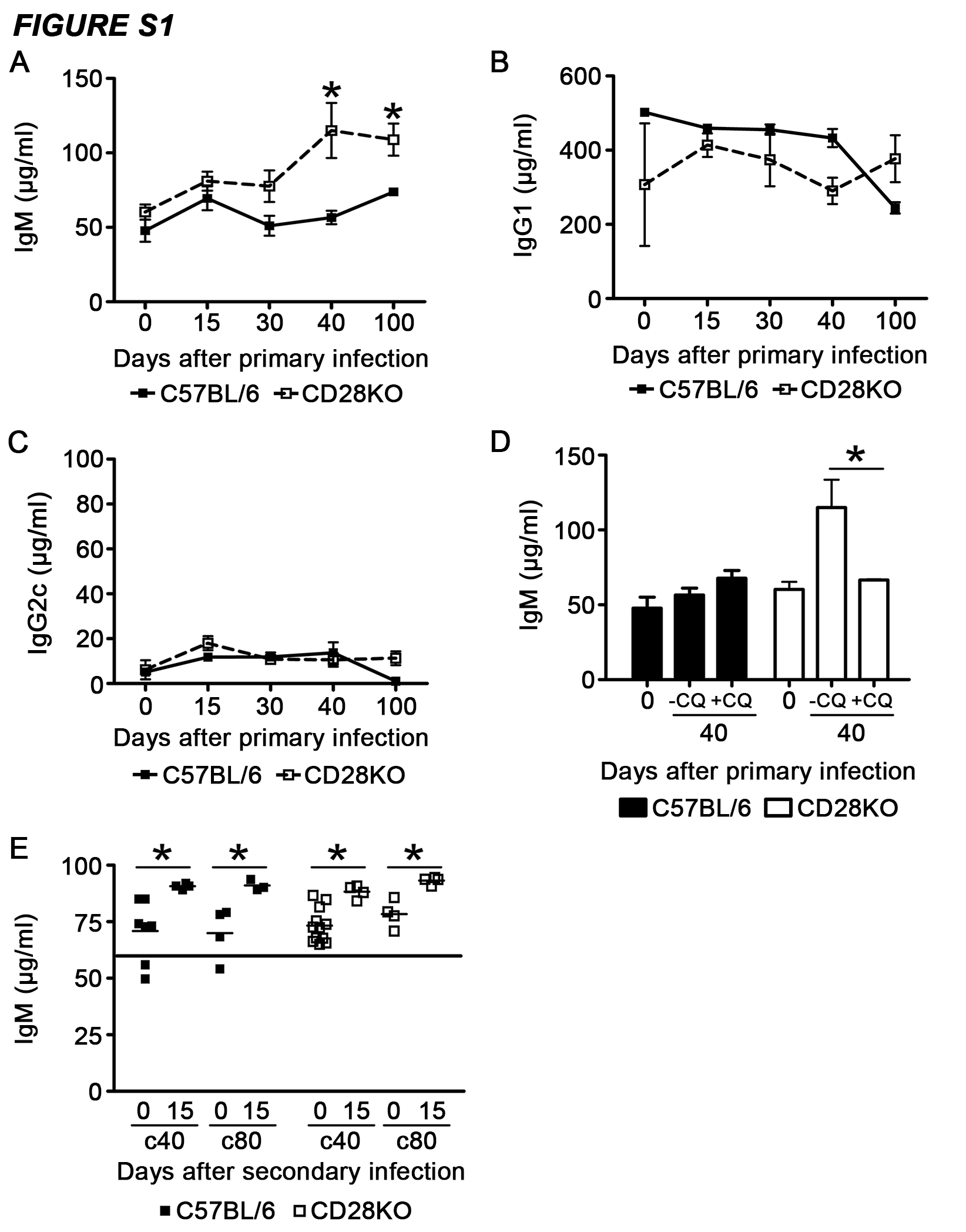

Supplement: S1 Fig — (a-c) Total IgM, IgG1 and IgG2c serum concentrations during primary infection. (d) Total IgM serum concentrations at day 40 p.i., in mice that were or were not treated with chloroquine (CQ). (e) Total IgM serum concentrations in c40 and c80 mice. In a-e, significant differences (p < 0.05) between the indicated groups are designated by *. One representative experiment out of three (n = 3–7, means ± SEM) is shown. (TIF) [file pone.0202522.s001.tif]

a

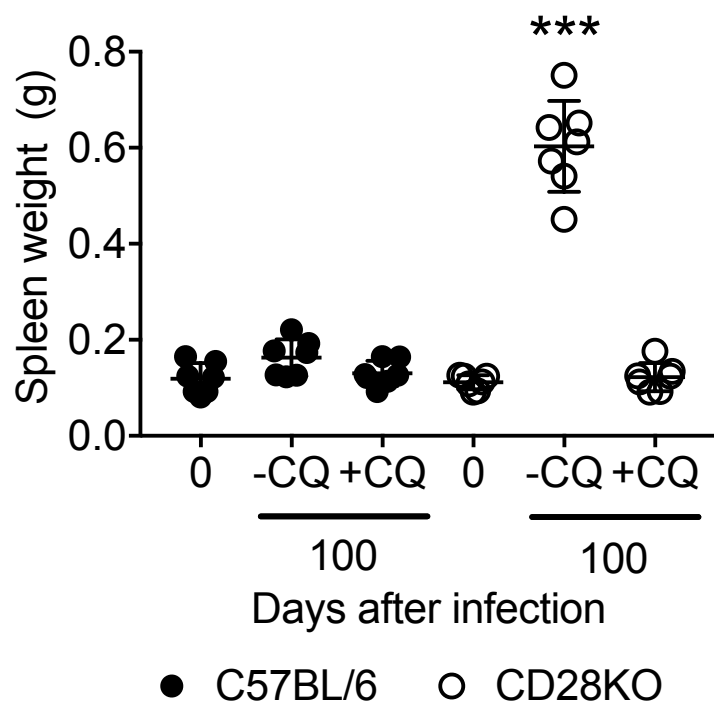

b

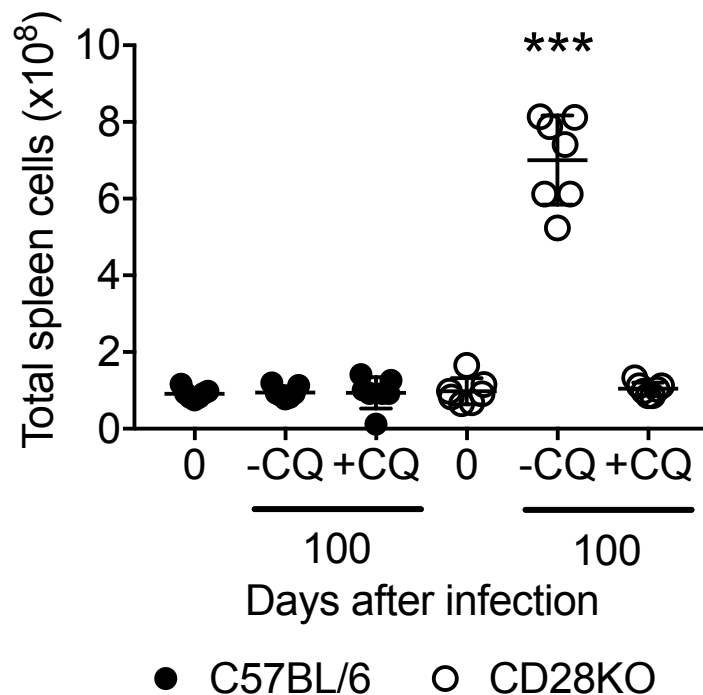

c

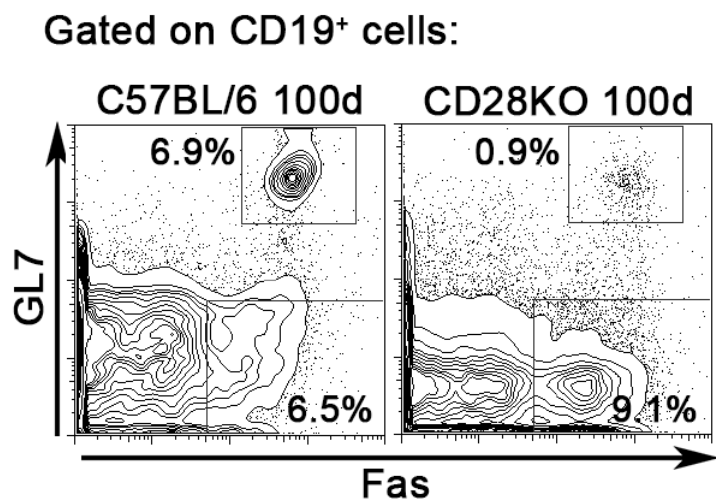

d

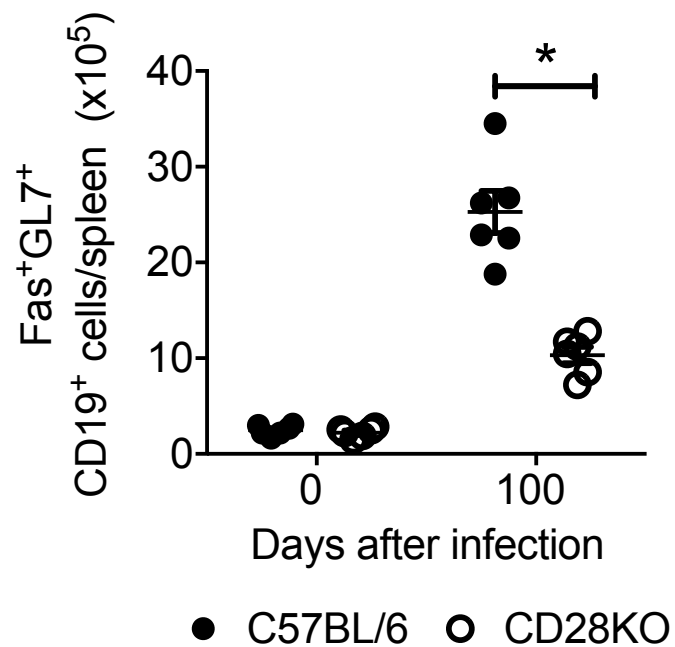

Supplement: S2 Fig — (a-d) Mice on day 30 p.i., were treated or not with chloroquine (+CQ) to eliminate reminiscent parasitemia or not (-CQ) and analyzed on day 100 p.i. (a) Data showing spleen weights. (b) Data showing total numbers of spleen cells. (c) Representative contour plots obtained by flow cytometry showing Fas and GL7 expression in CD19+ cells. The Fas+GL7+ and Fas+GL7- cell percentage data are shown. (d) The Fas+GL7+CD19+ cell numbers per spleen. In a-d, significant differences (*p<0.05, **p<0.01, ***p<0.001) between all experimental groups (C57BL/6 and CD28KO) are shown. Data from three independent experiments (n = 6–7, means ± SEM) is shown. (PDF) [file pone.0202522.s002.pdf]
